# ALKBH7 and NLRP3 Co-Expression: A Potential Prognostic and Immunometabolic Marker Set in Breast Cancer Subtypes

**DOI:** 10.3390/ijms262311661

**Published:** 2025-12-02

**Authors:** Adem Senturk, Nur Kazan, Selen Sen, Gozde Cakirsoy Cakar, Lacin Tatli Ayhan, Fuldem Mutlu, Onur Taydas, Barıs Mantoglu, Yasemin Gunduz, Metin Ercan, Zulfu Bayhan, Emine Yildirim, Hafize Uzun

**Affiliations:** 1 Department of Surgical Oncology, Sakarya University, Sakarya Training and Research Hospital, 54100 Sakarya, Turkey; ercan@sakarya.edu.tr; 2 Department Field Crops Division, Graduate Education Institute, Sakarya University of Applied Sciences, 54100 Sakarya, Turkey; nur.kazan@ogr.sakarya.edu.tr; 3 Department of Pharmacy Services, Pamukova Vocational School, Sakarya University of Applied Sciences, 54100 Sakarya, Turkey; scakirsoy@subu.edu.tr; 4 Department of Medical Pathology, Faculty of Medicine, Sakarya University, 54100 Sakarya, Turkey; gozdec@sakarya.edu.tr; 5 Department of Internal Medical Sciences, Faculty of Medicine, Sakarya University, 54100 Sakarya, Turkey; lacintatli@sakarya.edu.tr (L.T.A.); mutlufuldem@gmail.com (F.M.); taydasonur@gmail.com (O.T.); ygunduz@sakarya.edu.tr (Y.G.); 6 Department of General Surgery, Faculty of Medicine, Sakarya University, 54100 Sakarya, Turkey; barsm@sakarya.edu.tr (B.M.); zulfubayhan@sakarya.edu.tr (Z.B.); 7 Department of General Surgery, Faculty of Medicine, Istanbul Atlas University, 34403 Istanbul, Turkey; emine.yildirim@atlas.edu.tr; 8 Department of Medical Biochemistry, Faculty of Medicine, Istanbul Atlas University, 34403 Istanbul, Turkey; huzun59@hotmail.com

**Keywords:** breast cancer, ALKBH7, ALKBH7, HER2+, triple-negative breast cancer, Ki-67, Luminal A, Luminal B

## Abstract

Breast cancer (BC) is a heterogeneous disease with distinct molecular subtypes that exhibit variable immune responses and metabolic profiles. Recent studies have suggested that immunometabolic pathways play a role in tumor progression and treatment resistance. This study investigates the expression patterns of ALKBH7 and NLRP3 across BC molecular subtypes and explores their relationships with clinicopathological parameters and potential immunometabolic profiles. A total of 118 BC patients were classified into HER2+, TNBC, Luminal A, and Luminal B subtypes. Gene expression levels of ALKBH7 and NLRP3 were analyzed using quantitative real-time PCR (qRT-PCR), and correlations with clinical markers were assessed. ALKBH7 and NLRP3 expression levels varied significantly between subtypes, with the highest expression observed in HER2+ tumors. Strong positive correlations were found between ALKBH7 and NLRP3 in all subtypes, particularly in HER2+ (r = 0.812, *p* < 0.001). Additionally, NLRP3 correlated with Ki-67 in Luminal B tumors, indicating a link between inflammation and proliferative capacity. These findings suggest that ALKBH7 may function as a dual-role biomarker involved in metabolic adaptation and immune signaling in BC. The strong co-expression of ALKBH7 and NLRP3 suggests a functional association between these molecules that may be critical in shaping the tumor microenvironment. This co-expression set, particularly in aggressive subtypes (HER2+ and TNBC), warrants further mechanistic validation as a potential prognostic marker and a novel therapeutic vulnerability.

## 1. Introduction

Breast cancer (BC) is a heterogeneous disease classified into four subtypes: Luminal A, Luminal B, human epidermal growth factor receptor 2 (HER2)-enriched, and triple-negative, based on biomarker expression [1,2]. Despite improvements in early diagnosis and standard therapies, survival rates remain low in recurrent cases. Although molecularly targeted therapies show promise, their effectiveness is limited by the dynamic metabolic profiles of tumors. Therefore, novel therapeutic approaches targeting the tumor microenvironment (TME) and altered survival pathways are urgently needed.

A combination of molecularly targeted therapy with immunotherapy is a promising approach for advanced BC [3,4]. The TME promotes immune evasion by inducing immunosuppressive cells such as regulatory T cells (Tregs), M2 macrophages, and myeloid-derived suppressor cells (MDSCs). These cells secrete immunosuppressive cytokines (IL-10, TGF-β) and reactive oxygen species (ROS), enhancing tumor invasiveness and shaping a pro-tumor inflammatory milieu [5]. Such immune alterations facilitate tumor progression and metastasis. Thus, targeting TME-associated immunosuppression may improve immunotherapy efficacy and alter tumor dynamics.

Effective immunotherapy in BC requires the regulation of inflammation within the tumor microenvironment (TME). The NLR family pyrin domain containing 3 (NLRP3) inflammasome, known as a multimeric cytosolic protein complex, assembles within the cell in direct response to cellular stress and perturbations. The NLRP3 inflammasome, a key mediator of IL-1β secretion, has been implicated in BC progression, but its molecular role remains incompletely understood [6,7,8]. As a member of the AlkB family, AlkB homolog 7 (ALKBH7) has been demonstrated in multiple studies to participate in biological processes such as lipid metabolism and programmed necrosis. ALKBH7 is uniquely localized to the mitochondrial matrix and is involved in alkylation-oxidation-induced cell death [9]. Unlike ALKBH2/3, which directly repairs DNA damage, ALKBH7 contributes to cellular stress responses via mitochondrial pathways [10,11,12].

Emerging studies associate high ALKBH7 expression with poor prognosis, potentially due to enhanced M2 macrophage infiltration and ROS accumulation, which impair dendritic cell function and support immune evasion [13,14]. These findings position ALKBH7 as a potential therapeutic target involved in both metabolic regulation and immune modulation within the TME. The NLRP3 inflammasome drives IL-1β/IL-18 release and pyroptosis, shaping innate immunity. In cancer, it shows dual roles, enhancing antitumor responses or promoting chronic inflammation and immune evasion [6,7,8].

In BC, this context-dependent function underscores the importance of investigating ALKBH7’s potential interaction with NLRP3. Notably, the role of ALKBH7 in immune-mediated tumor progression in BC has not been previously characterized. The present study aimed to explore the transcriptional relationship between ALKBH7 and NLRP3 across molecular subtypes of BC, providing hypothesis-generating insights for future functional validation. The evidence of ALKBH7 and NLRP3 co-expression proposes a novel functional link between these molecules, potentially representing a dual-function mechanism that integrates metabolic adaptation and immune signaling in breast cancer.

These findings provide new insights into immunometabolic therapeutic approaches for aggressive and treatment-resistant breast cancer subtypes such as HER2+, Luminal B, and triple-negative breast cancer (TNBC).

## 2. Results

### 2.1. Comparison of Clinicopathological Data in Patient Groups

The control group consisted of fibroadenoma samples from 33 individuals, serving as non-malignant comparators. Table 1 summarizes the molecular subtypes and clinicopathological features. Mean age differed significantly across subtypes (*p* = 0.003), being highest in HER2+ (50.1 ± 6.9 years) and lowest in TNBC (42.6 ± 9.3 years). Ki-67 index varied significantly (*p* < 0.001), with TNBC showing the highest proliferation (50.8 ± 7.5), followed by HER2+ (40.5 ± 8.2), and lower levels in Luminal A (15.3 ± 5.4) and Luminal B (25.7 ± 6.1). ER and PR expressions varied significantly among the molecular subtypes (*p* < 0.001), showing universal positivity in luminal tumors. The HER2-positive group, defined inclusively, contained cases with co-expressed HRs (ER: 82.4%, PR: 79.4%). In our cohort, tumors with ER and PR ratios below 10% were classified within the TNBC group, as they fell into the ‘low positive’ range and behaved similarly to triple-negative disease biologically. Analysis of the entire cohort revealed that HER2 scores varied significantly across the subtypes (*p* < 0.001), with strong positivity (3+) characterizing 85.3% of the HER2-positive group.

### 2.2. Comparison of Gene Expression Levels of Groups

**Figure 1:**
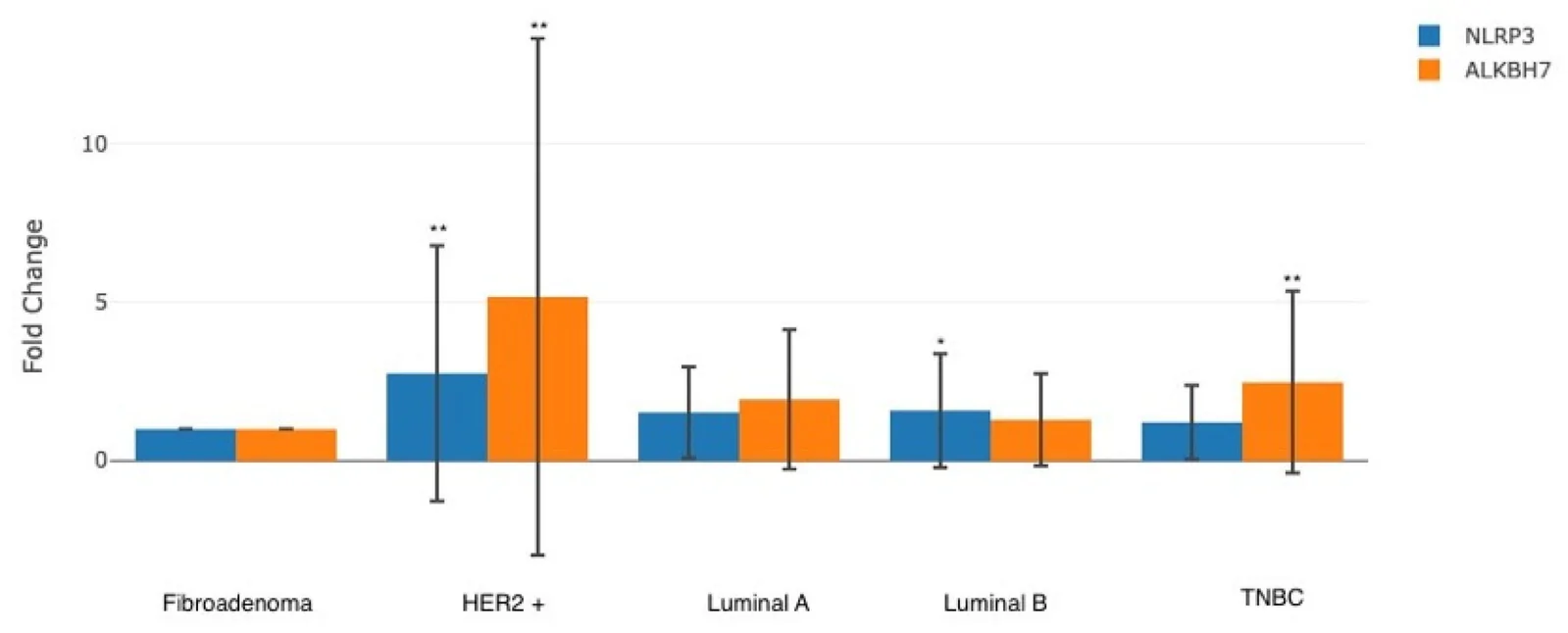
Expression profiles of ALKBH7 and NLRP3 in breast tumors and fibroadenomas (* *p* < 0.05, ** *p* < 0.01).

Figure 1 and Table 2 show the ALKBH7 and NLRP3 expression across groups. ALKBH7 levels differed significantly (*p* < 0.001), with HER2+ tumors expressing the highest levels, while TNBC exceeded Luminal B (*p* = 0.045). NLRP3 also varied (*p* = 0.004), being higher in HER2+ compared with Luminal A (*p* = 0.030) and TNBC (*p* = 0.005). TNBC showed the lowest NLRP3 expression.

### 2.3. Relationship Between Gene Expression and Clinical Data in Groups

ALKBH7 and NLRP3 were positively correlated across all subtypes, most strongly in HER2+ (r = 0.812, *p* < 0.001) (Table 3). ALKBH7 was unrelated to age, but NLRP3 correlated positively (r = 0.243, *p* = 0.003). Table 4 shows the correlation analysis of gene expressions in groups.

### 2.4. Comparison of Gene Expression Correlation Between Groups

As shown in Table 5, ALKBH7 and NLRP3 expression varied by subtype and clinicopathological features (*p* < 0.001). The relationship between ALKBH7 and Ki-67 varied among molecular subtypes: a positive correlation was observed in the Luminal A (r = 0.560, *p* < 0.001) and Luminal B (r = 0.511, *p* < 0.001) tumors, whereas a negative correlation was found in the HER2+ (r = −0.457, *p* = 0.003) and TNBC (r = −0.313, *p* = 0.006) subtypes (Table 5 and Table 6). HER2+ tumors displayed strong negative correlations with ER, PR, and Ki-67, while TNBC consistently showed the lowest expression. Luminal subtypes demonstrated higher levels overall, with Luminal A exhibiting particularly strong associations with ER and PR.

When analyzed across all samples, ALKBH7 showed a weak negative correlation with Ki-67 (r = −0.276, *p* = 0.003). However, subtype-specific analysis revealed distinct patterns: ALKBH7 was positively correlated with Ki-67 in the Luminal A (r = 0.560, *p* < 0.001) and Luminal B (r = 0.511, *p* < 0.001) tumors, while a negative correlation was observed in the HER2+ (r = −0.457, *p* = 0.003) and TNBC (r = −0.313, *p* = 0.006) subtypes. These findings indicate that the association between ALKBH7 and proliferative activity is subtype dependent.

## 3. Discussion

This study investigated the differential expression of ALKBH7 and NLRP3 across molecular subtypes of BC and examined their associations with clinicopathological characteristics and potential immunometabolic interactions. Our findings revealed that the ALKBH7 and NLRP3 gene expressions differed significantly among BC molecular subtypes, with the HER2+ group exhibiting the highest levels of both markers. This suggests a potential link between HER2 signaling and enhanced immunometabolic activity. Notably, the positive correlation between ALKBH7 and NLRP3 across all subtypes was particularly strong in the HER2+ group, highlighting their coordinated involvement in regulating inflammation and mitochondrial stress responses. The association of NLRP3 with Ki-67 in Luminal B tumors further implicates inflammasome activation in proliferative dynamics. These results support the hypothesis that ALKBH7 may contribute to tumor progression by influencing mitochondrial metabolism and inflammation, positioning it as a potential dual therapeutic target in BC.

Various studies indicate that ALKBH7, which belongs to the AlkB family, assumes a regulatory role in critical biological processes such as lipid metabolism and programmed necrosis [9,12]. A multimeric protein complex located in the cytosol, the NLRP3 inflammasome, is characterized by its assembly following various forms of cellular perturbation [8]. Our findings demonstrate that ALKBH7 and NLRP3 expression levels differ significantly among BC subtypes, with coordinated upregulation most evident in HER2+ tumors, suggesting an immunometabolic link. Their strong correlation supports a joint role in inflammation and cellular stress, while the association between NLRP3 and Ki-67 in Luminal B tumors indicates involvement in tumor proliferation. Overall, these results highlight ALKBH7 as a potential therapeutic target through its effects on mitochondrial metabolism and immune regulation.

This study included only female participants to eliminate gender-related gene expression variability. TNBC and HER2+ subtypes displayed aggressive features with high Ki-67, while Luminal A showed favorable markers (low Ki-67, high ER/PR), consistent with prior studies [15,16]. Given the link between immunotherapy resistance and aggressive features like HER2 overexpression and proliferation [3,4], combining HER2-targeted and immunotherapies may benefit HER2+ patients [16]. We observed significantly higher ALKBH7 and NLRP3 expression in HER2+ tumors, indicating an inflammatory profile. This aligns with evidence on NLRP3 inflammasome activation in tumor progression via IL-1β and IL-18 maturation [17]. Our findings also support Chen et al. [18], who identified ALKBH7 as a poor prognostic marker associated with immune modulation in BC. The strong correlation between ALKBH7 and NLRP3 in HER2+ tumors suggests a role for ALKBH7 in immunometabolic regulation and immune evasion, reinforcing its potential as a dual prognostic and therapeutic target.

Tumor-associated macrophage (TAM) derived cytokines such as VEGF and CCL8 promote angiogenesis and tumor progression [19,20], while NLRP3/IL-1β expression in TAMs is associated with reduced survival and increased lymph node metastasis in HER2+ patients [8,17,19]. In our study, ALKBH7 and NLRP3 expression, upregulated in HER2+ and TNBC, showed a negative correlation with Ki-67, suggesting a shift toward an immune evasion phenotype rather than proliferation. Especially in HER2+ and TNBC, ALKBH7 may promote mitochondrial stress resistance over cell division. This is consistent with Wang et al. [21], who linked high ALKBH7 expression to poor prognosis and immune suppression in HNSC. While their study emphasized pan-cancer implications, ours reveals a subtype-specific role in BC, particularly in HER2+, highlighting ALKBH7’s relevance in immunometabolic regulation. Furthermore, Wu et al. [22] showed that PRMT5–ALKBH5 interaction regulates doxorubicin response via RNA demethylation and DNA repair in BC. Together with our findings on ALKBH7, this suggests that AlkB family proteins influence tumor metabolism, immune escape, and therapy resistance, positioning them as promising therapeutic targets across cancer types.

In this study, the positive correlation between ALKBH7 and NLRP3 suggests a link between mitochondrial stress and inflammation, contributing to immune suppression in the tumor microenvironment. While proliferative tumors rely on glycolysis (Warburg effect), elevated ALKBH7 expression despite low Ki-67 implies a metabolic shift toward OXPHOS, often associated with low proliferation but high resistance. This shift may increase ROS production, a well-known activator of the NLRP3 inflammasome, promoting immune evasion and chronic inflammation. In HER2+ and TNBC tumors, ALKBH7-driven mitochondrial stress likely enhances NLRP3 activation, creating an immunosuppressive microenvironment by inhibiting T cell responses [13,23,24]. Thus, ALKBH7 may facilitate tumor survival via OXPHOS-induced inflammation and immune modulation.

Rather than triggering immune responses through excessive proliferation, tumor cells may develop resistance to immunotherapy by shifting toward mitochondrial stress, mediated by ALKBH7. This stress increases cytochrome C release, promoting intrinsic apoptosis. However, ALKBH7 appears to counterbalance this risk via NLRP3, which activates survival pathways (NF-κB, STAT3) and suppresses apoptosis by inhibiting caspase activity through caspase-1. This ALKBH7–NLRP3 co-expression may enable tumor cells, particularly in aggressive BC subtypes, to resist immune-mediated death, fostering immune-resistant phenotypes within the tumor microenvironment. By neutralizing ROS-induced apoptotic cascades, high ALKBH7 and NLRP3 levels may predict poor response to immunotherapy [23,24,25,26,27].

The correlation analysis revealed a context-dependent relationship between ALKBH7 and Ki-67. Specifically, ALKBH7 expression was positively correlated with proliferative activity in Luminal A and Luminal B tumors but negatively correlated in HER2+ and TNBC subtypes. This duality suggests that ALKBH7 may exert divergent biological functions depending on the molecular background of the tumor. In hormone receptor–positive (Luminal) cancers, ALKBH7 may support cell proliferation by maintaining mitochondrial homeostasis and metabolic flexibility, whereas in more aggressive HER2+ and TNBC subtypes, it may promote cellular adaptation to stress through enhanced oxidative phosphorylation and the suppression of apoptosis. These contrasting roles highlight the complex contribution of ALKBH7 to BC progression and its potential as a context-specific biomarker of tumor behavior.

The structural study by Wang et al. [28] provided key insights into the distinct biochemical properties of ALKBH7, highlighting its role in programmed necrosis and fat metabolism, unlike other AlkB homologs that primarily repair nucleic acids. Their findings demonstrated that ALKBH7 lacks a nucleotide-binding domain and likely targets protein substrates, suggesting a unique functional role within the AlkB family. In line with this, our results support a non-canonical role for ALKBH7 in BC, particularly through its involvement in mitochondrial metabolism and immune modulation. The high ALKBH7 expression observed in HER2+ tumors and its correlation with NLRP3 inflammasome activation imply that ALKBH7 may influence breast carcinogenesis not through direct DNA repair, but via metabolic reprogramming and the regulation of immune responses. These complementary findings strengthen the hypothesis that ALKBH7 functions as a critical immunometabolic mediator, with structural features that may be exploited for therapeutic targeting of cancer.

It is worth noting that the present study was designed as an observational and correlative analysis based on gene expression data. Although a strong association between ALKBH7 and NLRP3 expression was identified, functional validation through gene knockdown, overexpression, or knockout models was beyond the current study’s scope. Future mechanistic studies are needed to confirm the causal relationship and clarify how the ALKBH7–NLRP3 co-expression contributes to immune modulation and tumor progression in BC.

To clarify, our use of the term *immune profile* refers to an inferred immunometabolic association rather than direct immune profiling. This inference was based on previous evidence linking NLRP3 inflammasome activation and ALKBH7-mediated mitochondrial stress to immune modulation within the tumor microenvironment [13,21]. To avoid overinterpretation, we replaced the term immune profile with potential immunometabolic association throughout the manuscript. In future studies, we plan to perform immune profiling and cytokine quantification (IL-1β, IL-18, TNF-α) to experimentally validate these interactions.

These results support the hypothesis that ALKBH7 may contribute to tumor progression by influencing mitochondrial metabolism and inflammation, positioning it as a potential dual therapeutic target in BC.

Interestingly, the relationship between ALKBH7 expression and Ki-67 was not uniform across molecular subtypes. As shown in Table 5, Luminal A and Luminal B tumors displayed a positive correlation, whereas HER2+ and TNBC subtypes exhibited a negative correlation. This pattern suggests that the biological role of ALKBH7 in regulating proliferative activity is context dependent. In Luminal subtypes, where metabolic flexibility supports hormone receptor-driven proliferation, ALKBH7 expression may facilitate energy adaptation and cell cycle progression. Conversely, in aggressive HER2+ and TNBC subtypes, elevated ALKBH7 may instead contribute to mitochondrial stress tolerance and immune evasion, rather than promoting proliferation. These subtype-specific differences highlight that ALKBH7’s function may shift from a metabolic facilitator in hormone-responsive tumors to a stress adaptation and survival mediator in high-risk subtypes, aligning with its known roles in mitochondrial regulation and cellular resilience.

### Limitations and Future Directions

Although this study provides important insights into the transcriptional relationship between ALKBH7 and NLRP3, it is based solely on RNA-level analyses. Therefore, the observed associations between gene expression and clinicopathological or immunometabolic parameters should be interpreted as correlative rather than causative. Future research will aim to validate these findings through protein-level analyses (e.g., Western blot, immunohistochemistry) and functional experiments such as siRNA-mediated knockdown and co-culture assays with immune cells to better define the mechanistic role of the ALKBH7–NLRP3 co-expression in BC.

This study also has several limitations. It did not evaluate long-term clinical outcomes such as survival or treatment response. Potential confounders, including prior therapies, comorbidities, and lifestyle factors, were not controlled, which may influence gene expression and the immune microenvironment. Moreover, tumor microenvironmental heterogeneity and inter-individual immune variability were not fully addressed. The relatively small sample size, especially within subgroups, may also limit statistical power and generalizability. Despite these limitations, our findings provide valuable preliminary evidence for the immunometabolic role of ALKBH7 and NLRP3 in BC and warrant further validation in larger, prospective, and mechanistic studies.

These findings suggest that ALKBH7 may serve as a dual-function biomarker, influencing both metabolic adaptation and immune regulation in breast cancer. By modulating cellular metabolism and immune cell infiltration, ALKBH7 may impact tumor progression and treatment response. Its coordinated expression with NLRP3, particularly in HER2+ tumors, points to a potential ALKBH7–NLRP3 co-expression that could shape the tumor microenvironment dynamics and serve as a prognostic biomarker or therapeutic target, especially in aggressive subtypes such as HER2+ and TNBC. Future work involving gene silencing, knockout (KO), or overexpression models is planned to validate the mechanistic role of ALKBH7–NLRP3 co-expression in regulating tumor–immune interactions.

## 4. Materials and Methods

### 4.1. Selection of the Study Group

The current study included tru-cut biopsy samples taken from patients at the time of diagnosis between 1 January 2024, and 1 January 2025, at the Department of Surgical Oncology at Sakarya Training and Research Hospital in Turkey. Patients with pathological, clinical, and radiological evidence that the tumor had come to the breast through direct invasion or metastasis from another organ tumor were not included in the groups formed by histological/pathological confirmation. The control group was composed of benign fibroadenoma breast tissues.

This study utilized the G*Power 3.1.9.7 software to determine the appropriate sample size for comparing ALKBH7 and NLRP3 gene expression levels across different BC molecular subtypes, including Luminal A, Luminal B, HER2+, and triple negative. For ALKBH7, an effect size of f = 0.796 was calculated using a one-way ANOVA (fixed effects, omnibus, one-way), indicating that a minimum of 7 participants per group would be sufficient to achieve 80% statistical power (1 − β = 0.80). For NLRP3, the estimated effect size was f = 0.456, and to reach a comparable power level, at least 14 participants per group was deemed necessary. A significance level of α = 0.05 was applied in all calculations.

Tumor molecular subtypes were classified according to the 2013 St. Gallen Consensus criteria, based on ER, PR, HER2, and Ki-67 immunohistochemical profiles (2). The study included a total of 118 BC patients, classified into molecular subtypes based on immunohistochemical biomarker profiles: HER2-positive (n = 34), TNBC (n = 22), Luminal A (n = 34), and Luminal B (n = 28). The control group consisted of benign fibroadenoma breast tissue samples obtained from 33 individuals, serving as non-malignant comparators for gene expression analysis.

An immunohistochemical staining procedure was applied with Ki-67 (SP6) (Neomarkes, Oviedo, FL, USA) ready-to-use rabbit monoclonal antibody.

### 4.2. RNA Extraction from Biopsy Tissue Samples

Tru-cut biopsy samples were collected in sterile Eppendorf tubes and placed in Hank’s Balanced Salt Solution (HBSS) containing 1% Penicillin-Streptomycin. Tissue fragments were then treated with Proteinase K (Genaxxon Bioscience™, Ulm, Germany) and incubated overnight at 55 °C in a heat block. Following incubation, the samples were heated at 90 °C for 30 min to inactivate Proteinase K. Subsequently, TriGent reagent was added according to the manufacturer’s protocol. For every 1 mL of reagent, 200 μL of chloroform (Sigma™, St. Louis, MO, USA) was added. The mixture was centrifuged at 12,000 rpm at 4 °C for 15 min. The upper aqueous phase containing RNA was carefully collected, and RNA was precipitated using isopropanol. This was followed by a second centrifugation at 12,000 rpm at 4 °C for 10 min to obtain the RNA pellet. The pellet was washed with 70% ethanol, and residual ethanol was evaporated at room temperature. Diethylpyrocarbonate (DEPC)-treated water was then added to dissolve the RNA, followed by incubation at 65 °C for 15 min. The purified RNA samples were stored at −80 °C until further use. RNA concentrations were measured using a NanoDrop 2000C spectrophotometer (ThermoFisher Scientific, Waltham, MA, USA).

### 4.3. Gene Expression Analysis via Quantitative Real-Time PCR (qRT-PCR)

mRNA expression levels of Takeda G protein-coupled receptor 5 (TGR5) and NADPH Oxidase 5 (NOX5) were analyzed using qRT-PCR. The primers used were as follows:

- **ALKBH7**: Sense:5′-GGAGCCAGATGTTGAGAG-3′Antisense: 5′-CTGAGGCTACAATTCCAGGTC-3′
- **NLRP3**: Sense: 5′-CAGCCTCATCAGAAAGAAGC-3′Antisense: 5′-GTGCTGCAGTTTCTCCAGG-3′
- **GAPDH (reference gene)**: Sense: 5′-CTTCCTGAGCCTACTGCTGG-3′Antisense: 5′-AGTCGAAGTTGAGGCACTGG-3′

Each qPCR reaction consisted of 4 μL of cDNA, 0.8 μL of each forward and reverse primer, 10 μL of SensiFAST™ SYBR^®^ No-ROX Kit (BioLine, Taunton, MA, USA), and 4.4 μL of DEPC-treated water. Reactions were run on the CFX Connect™ Real-Time PCR System (Bio-Rad, Feldkirchen, Germany). The cycling conditions were initial denaturation at 95 °C for 2 min, followed by 40 cycles of 95 °C for 5 s, 65 °C for 10 s, and 72 °C for 2 min. Gene expression levels were normalized to GAPDH, and relative quantification was calculated using the 2^−ΔΔCt^ method. Data visualization was performed using GraphPad Prism version 9.0.0.

#### Ethical Statement

This study was conducted in accordance with the ethical principles outlined in the Declaration of Helsinki and adhered to Good Clinical Practice (GCP) guidelines. Ethical approval was obtained from the Clinical Research Ethics Committee of Sakarya University (Approval Date: 5 September 2022; Decision No: 161889-109). Informed consent was obtained from all participants before their inclusion in the study.

### 4.4. Statistical Analysis

All statistical analyses were conducted using SPSS software (version 30.0). To ensure adequate statistical power, sample size calculations were performed with G*Power (version 3.1.9.6), determining the minimum required sample size at a 95% confidence level. Gene expression differences among molecular subtypes were analyzed using one-way ANOVA for normally distributed data and the Kruskal–Wallis test for non-parametric data. The Chi-square test was applied to assess associations between categorical variables. Spearman correlation analysis was used to evaluate relationships between gene expression levels and clinical parameters. Statistical significance was set at *p* < 0.05.

## Figures and Tables

**Table 1:** Clinicopathological features and treatment characteristics of patients stratified by breast cancer subtypes.

Variable		Fibroadenoma (n = 33)		HER2+ (n = 34)	TNBC (n = 22)			Luminal A (n = 34)		Luminal B (n = 28)		F Test	*p*-Value
		Mean ± SD		Mean ± SD	Mean ± SD			Mean ± SD		Mean ± SD			
**Age (years)**		43.9 ± 10.6		50.1 ± 6.9	42.6 ± 9.3			45.2 ± 8.1		47.8 ± 7.6		28.665	<0.001
**Ki-67**		-		40.5 ± 8.2	50.8 ± 7.5			15.3 ± 5.4		25.7 ± 6.1		26.620	<0.001
		**n**	**%**	**n**	**%**	**n**	**%**	**n**	**%**	**n**	**%**	***χ*^2^ test:**	***p*-value**
**ER Status**	**Positive**	0	0.0	28	82.4	1	4.5	34	100	28	100	259.245	*p* < 0.001
	**Negative**	0	0.0	6	17.6	21	95.5	0	0	0	0		
**PR Status**	**Positive**	0	0.0	28	82.4	11	50	34	100	27	96.4	189.099	*p* < 0.001
	**Negative**	0	0.0	6	17.6	11	50	0	0	1	3.6		
**CerbB2 Status**	**Score 0**	0	0.0	0	0	6	27.3	1	2.9	5	17.9	284.438	*p* < 0.001
	**Score 1**	0	0.0	0	0	1	4.5	2	5.9	7	25.0		
	**Score 2**	0	0.0	5	14.7	2	9.1	0	0	0	0		
	**Score 3**	**0**	0.0	29	85.3	13	59.1	31	92.1	16	57.1		

**F test:** One-way ANOVA test, ***χ*^2^ test:** Chi-square test, *p* < 0.05 statistical significance. **ER:** Estrogen Receptor, **PR:** Progesterone Receptor, **HER2:** Human Epidermal Growth Factor Receptor 2, **TNBC**: Triple-Negative Breast Cancer, **Ki-67:** Proliferation Index.

**Table 2:** Comparison of gene expression levels of groups.

Variable	Group	n	Mean	SD	F Test	*p*-Value
**ALKBH7 mRNA**	**Fibroadenoma**	33	-	-	**14.666**	<0.001 *
	**Luminal A**	34	0.9506	0.9007		
	**Luminal B**	28	0.3689	0.8536		
	**HER2+**	34	2.3679	1.8120		
	**TNBC**	22	1.3024	0.9578		
**NLRP3 mRNA**	**Fibroadenoma**	33	-	-	**4.757**	0.004 *
	**Luminal A**	34	0.6025	0.8091		
	**Luminal B**	28	0.6566	1.2108		
	**HER2+**	34	1.4617	1.7943		
	**TNBC**	22	0.2789	0.8285		

**F test:** One-way ANOVA test, * *p* < 0.05, statistical significance.

**Table 3:** Correlation analysis of gene expressions in groups.

Variable	Group		NLRP3 mRNA
**ALKBH7 mRNA**	**Luminal A**	r	0.346
		*p*	0.045 *
	**Luminal B**	r	0.568
		*p*	0.002 *
	**HER2+**	r	0.812
		*p*	0.001 *
	**TNBC**	r	0.454
		*p*	0.034 *

Pearson correlation, r: correlation coefficient, * *p* < 0.05 statistical significance.

**Table 4:** Correlation analysis of clinicopathological data.

Group		ALKBH7 mRNA	NLRP3 mRNA
**Age**	r	0.031	0.243
	*p*	0.710	0.003 *
**Ki-67**	r	−0.276	0.103
	*p*	0.003 *	0.275
**ER Status**	r	0.690	0.648
	*p*	0.040 *	0.042 *
**PR Status**	r	−0.967	−0.785
	*p*	0.004 *	0.025 *
**CerbB2 Status**	r	0.182	0.068
	*p*	0.049 *	0.466

Pearson correlation, r: correlation coefficient, * *p* < 0.05 statistical significance.

**Table 5:** Gene expression in groups and Pearson correlation with clinicopathological data.

		ALKBH7				NLRP3			
		Luminal A n = 34	Luminal B n = 28	HER2+ n = 34	TNBC n = 22	Luminal A n = 34	Luminal B n = 28	HER2+ n = 34	TNBC n = 22
**Age**	r	0.483	0.418	0.371	0.210	0.517	−0.470	0.434	0.223
	*p*	0.000 **	0.002 **	0.006 *	0.011 *	0.000 **	0.002 *	0.003 *	0.010 *
**Ki-67**	r^s^	0.560	0.511	−0.457	−0.313	0.644	0.559	−0.448	0.269
	*p*	0.000 **	0.000 **	0.003 *	0.006 *	0.000 **	0.000 **	0.003 *	0.016 *
**ER Status**	r	0.664	0.587	−0.663	0.508	−0.653	0.565	−0.659	0.442
	*p*	0.000 **	0.000 **	0.000 **	0.000 **	0.000 **	0.000 **	0.000 **	0.003 *
**PR Status**	r	0.754	0.668	−0.771	0.575	0.661	0.646	0.754	0.571
	*p*	0.000 **	0.000 **	0.000 **	0.000 **	0.000 **	0.000 **	0.000 **	0.000 **
**CerbB2 Status**	r	0.702	0.542	0.348	0.461	−0.596	−0.305	0.336	0.264
	*p*	0.000 **	0.000 **	0.006 *	0.003 *	0.000 **	0.007 *	0.006 *	0.014 *

r: Pearson correlation coefficient (** *p* < 0.001, * *p* < 0.05). r^s^: Spearman correlation.

**Table 6:** Gene expression in groups and Spearman correlation with clinicopathological data.

		ALKBH7				NLRP3			
		Luminal A n = 34	Luminal B n = 28	HER2+ n = 34	TNBC n = 22	Luminal A n = 34	Luminal B n = 28	HER2+ n = 34	TNBC n = 22
**Age**	r	−0.181	0.205	−0.269	−0.267	−0.014	−0.070	−0.274	−0.205
	*p*	0.305	0.296	0.124	0.229	0.937	0.725	0.117	0.361
**Ki-67**	r^s^	−0.136	0.855	−0.109	−0.027	0.079	0.361	−0.039	0.188
	*p*	0.442	0.037	0.539	0.911	0.656	0.064	0.829	0.428
**ER Status**	r	0.135	0.071	−0.069	0.031	−0.207	0.065	0.882	0.263
	*p*	0.158	0.118	0.696	0.891	**0.027**	0.489	**0.014**	0.237
**PR Status**	r	0.108	0.046	−0.069	0.054	0.123	0.166	0.068	0.373
	*p*	0.245	0.815	0.696	0.812	0.186	0.399	0.703	0.087
**CerbB2 Status**	r	0.053	−0.255	0.172	0.057	−0.059	−0.300	0.016	0.154
	*p*	0.767	0.191	0.330	0.799	0.739	0.121	0.929	0.493

r: Pearson correlation coefficient, r^s^: Spearman correlation.

## Data Availability

The original contributions presented in this study are included in the article. Further inquiries can be directed to the corresponding author.
